# Theoretically predicting the feasibility of highly-fluorinated ethers as promising diluents for non-flammable concentrated electrolytes

**DOI:** 10.1038/s41598-020-79038-y

**Published:** 2020-12-15

**Authors:** Amine Bouibes, Soumen Saha, Masataka Nagaoka

**Affiliations:** 1 Graduate School of Informatics, Nagoya University, Furo-cho, Chikusa-ku, Nagoya, 464-8601 Japan; 2 Elements Strategy Initiative for Catalysts and Batteries (ESICB), Kyoto University, Kyodai Katsura, Nishikyo-ku, Kyoto, 615-8520 Japan

**Keywords:** Chemistry, Electrochemistry, Physical chemistry, Theoretical chemistry

## Abstract

The practical application of nonflammable highly salt-concentrated (HC) electrolyte is strongly desired for safe Li-ion batteries. Not only experimentalists but also theoreticians are extensively focusing on the dilution approach to address the limitations of HC electrolyte such as low ionic conductivity and high viscosity. This study suggests promising highly-fluorinated ethers to dilute the HC electrolyte based on non-flammable trimethyl phosphate (TMP) solvent. According to the quantum mechanical and molecular dynamics calculations, the fluorinated ether diluents showed a miscibility behavior in HC TMP-based electrolyte. While such miscibility behavior of the diluent with TMP solvent has been significantly enhanced by increasing its degree of fluorination, i.e., the “fluorous effect”, it is remarkable that the self-diffusion constant of Li^+^ and the ionic conductivity should be significantly improved by dilution with bis(1,1,2,2-tetrafluoro ethyl) ether (B2E) and bis(pentafluoro ethyl) ether (BPE) compared to other common hydrofluoroether diluents. In addition, the fluorinated-ether diluents have high ability to form a localized-concentrated electrolyte in HC TMP-based solution, leading to high expectation for the formation of a stable and a compact inorganic SEI film.

## Introduction

The current researches concerning energy storage aim greatly to increase the energy and power density of Li-ion batteries (LIB) for wider applications while ensuring their safety^1,2^. Intense effort has been devoted recently to introduce nonflammable solvents, such as organic phosphates, using the approach of highly salt-concentrated (HC) electrolytes^2–4^. This new approach has shown not only an attractive nonflammability property but also an excellent charge–discharge performance comparable or superior to that of the conventional flammable carbonate electrolytes^4^. Actually, the increase in salt concentration decreased the free solvent concentration and hence decreased the mobility of electrolyte. In addition, the salt anions were mainly in aggregated state at HC electrolyte. As the change in the HC electronic structure, the location of the larger amplitude in the lowest unoccupied molecular orbital (LUMO) shifts from the solvent towards the aggregated-salt, resulting in the earlier reductive decomposition of the salt before the solvent at low potential^4,5^. Hence, the solid electrolyte interphase (SEI) layer was mainly formed from the reduction of aggregated salt anions^4–6^. Recently, it was revealed using Red Moon methodology^6–8^ that a large amount of salt anions is localized on the SEI surface in HC electrolyte, enhancing the network formation of a dense inorganic layer with SEI salt-derived species^6^. The formation of such a pure inorganic SEI layer, therefore, should considerably improve the stability of SEI layer and would bring about a longer lifetime of advanced safe LIB. Moreover, it was observed that the formation of localized aggregated salt anions is highly expected for a more compact and stable SEI layer leading to a high cycling performance of LIBs^3,9^.

However, the practical application of nonflammable HC electrolyte still suffers from some limitations as the low ionic conductivity and the high viscosity of the electrolyte^4,5^. In challenges to overcome these drawbacks, some researchers are focusing on the promising approach of dilution^9–17^. In fact, the selection and designing of diluents are very important to improve transports properties of electrolyte anions without losing their nonflammability characteristic and the formation of stable salt-derived SEI film^11,12^. Among them, hydrofluoroethers (HFEs) were widely used as diluent co-solvents^9,10,12–17^ because of their low solvating ability^9,10,14,15^. HFEs showed a high ability to enhance the oxidative stability of the electrolytes^13^, excellent thinning reagents for reducing the viscosity of electrolytes^14^ and to construct a localized-concentrated electrolyte, which is strongly requested for the formation of a stable salt-derived SEI film^9,12,16^. Regarding the general structure of HFEs, the common HFEs were classified according to the vicinity of the fluoroalkyl groups to the oxygen atom^17^. The HFEs, with the closest position of a fluoroalkyl group to the oxygen atom, showed the lowest lithium-solvating power, while the distant fluoroalkyl-substituted HFEs, as bis(2,2,2-trifluoroethyl) ether (BTFE), did the opposite effect in addition to the highest conductivity^17^. Also, ethyl 1,1,2,2-tetrafluoroethyl ether (ETE) showed a high ionic conductivity in addition to BTFE for acetonitrile-based electrolytes^10^. On the other hand, while a designing approach has been made in order to increase the degree of fluorination in HFEs, it was shown that the highly fluorinated HFEs, such as 1,1,2,2-tetrafluoroethyl 2,2,3,3-tetrafluoropropyl ether (TTE), yield much better capacity of retention and high charge–discharge cycling performance compared to that of less fluorinated HFE diluents^10,12^. However, it was also understood that such highly fluorinated HFEs did not show enough efficiency to significantly enhance the transport properties of electrolytes^10,12^.

## Results and discussion

**Figure 1:**
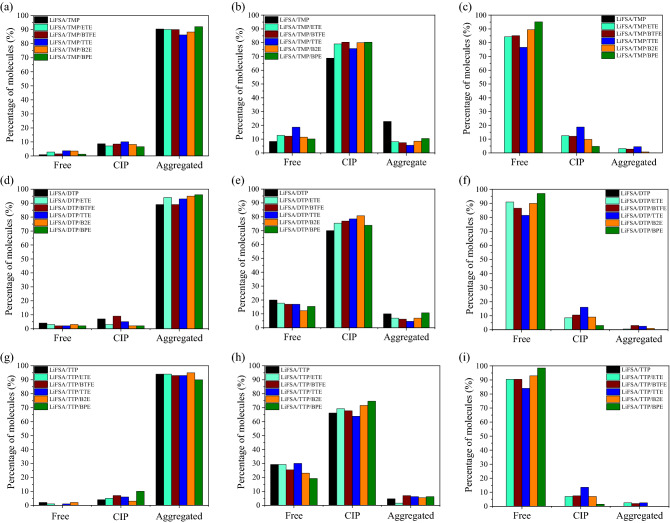
Averaged percentage of three main states of (**a**) salt anion, (**b**) TMP solvent and (**c**) ether diluents in TMP-based diluted electrolytes; (**d**) salt anion, (**e**) DTP solvent and (**f**) ether diluents in DTP-based diluted electrolytes; and (**g**) salt anion, (**h**) TTP solvent and (**i**) ether diluents in TTP-based diluted electrolyte solutions. The considered molar ratios were 1:1.3 for salt and solvent in HC electrolyte and 1: 1.3:2 for salt, solvent and diluents in the diluted electrolyte.

In this study, we investigated, microscopically, the dilution effect of the fire extinguishing HC electrolyte based on the nonflammable trimethyl phosphate (TMP) solvent and lithium bis(fluorosulfonyl)amide (LiFSA) salt^2–4^. The dilution effect has been examined by considering five different fluorinated ether molecules. Among them, three common HFEs (viz, ETE, BTFE, TTE), which have been actually studied^10,12,17^. In addition, we introduced, for the first time, two other fluorinated diethyl ether molecules, bis(1,1,2,2-tetrafluoro ethyl) ether (B2E) and bis(pentafluoro ethyl) ether (BPE) that consist of eight and ten fluorine atoms, respectively (Table 1). To understand the effect of each diluent molecule, we evaluated the solvation structure, the miscibility, and the transport properties using both molecular dynamics (MD) and quantum mechanical (QM) calculations (see Computational details). Herein, we referred to 1:1.3 LiFSA/TMP molar ratio as that of the HC electrolyte, and to 1:1.3:2 LiFSA/TMP/Diluents molar ratio as that of diluted electrolytes. Experimentally, this molar ratio showed an optimum diluent concentration for a maximum ionic conductivity of diluted LiFSA/TMP electrolyte by TTE^12^. Using MD simulations, the systems were mixed during 1 ns at 1000 K and relaxed at 298 K for 10 ns. The corresponding salt concentration of the present molar ratio was presented in Supplementary Table S1. First, we evaluated the solvation structure of HC and diluted electrolytes. Three main states of electrolyte compounds were considered: the free state, the contact-ion-pair (CIP) state, where the molecule is coordinated with one Li^+^, and the aggregate state, where the molecule is coordinated with two or more Li^+^. Indeed, the coordinated molecules with Li^+^ were estimated numerically by using the sum of the van der Waals radii of the atoms in a molecule and Li^+^. Figure 1 shows the averaged ratios over 10 samples of three main states of the FSA^-^ salt anion, TMP solvent, and ether diluents in HC electrolyte based on TMP solvent as well as in the diluted electrolytes. The ether molecules, especially BPE and B2E molecules, were mainly in their free states, due to their very weak interactions with Li^+^, − 7.87 and − 1.31 kcal/mol, respectively, compared to the stronger interaction between the TMP solvent and Li^+^, − 21.60 kcal/mol. In Fig. 1 (b), while the TMP solvent was mainly in CIP state, we can remark a clear decrease in the number of aggregated TMP by dilution and an evident increase of the CIP state rather than the Free-TMP due to the strong interaction between TMP and Li^+^ compared to those of different diluent ethers and Li^+^ (Table 2). Moreover, we can notice the formation of localized-concentrated electrolyte in such TMP-based electrolytes diluted with fluorinated-ethers. This result is in good agreement with experimental observations^9,10,12^.

**Table 1:** The considered systems used for this study.

Full name	Abbreviation	Atomic structure	Density (g/mL)	Boiling point (°C)
Ethyl 1,1,2,2 tetrafluoroethyl ether	ETE		1.204 (1.198^a^)	57
Bis(2,2,2-trifluoroethyl) ether	BTFE		1.398 (1.404^b^)	25
1,1,2,2- Tetrafluoroethyl 2,2,3,3-tetrafluoropropyl ether	TTE		1.536 (1.532^c^)	93.2
Bis(1,1,2,2-tetrafluoro ethyl) ether	B2E		1.552	–
Bis(pentafluoro ethyl) ether	BPE		1.562	–
Dimethyl(trifluoromethyl) phosphate	DTP		1.480	–
Tris(trifluoromethyl) phosphate	TTP		1.767	–

^a^Ref.^19^.

^b^Ref.^20^.

^c^Ref.^21^.

**Table 2:** The interaction energy (kcal/mol) between Li^+^, LiFSA and TMP with various systems (solvent or diluents) as well as the interaction energy (kcal/mol) between the diluents themselves (dimer) as obtained at M06-2X/def2-TZVP level in TMP (*ε* = 20.6) solvent.

System	Li^+^-system	LiFSA-system	TMP-system	Dimer
TMP	− 21.60 (− 19.38^a^)	− 18.36	− 5.77	−
ETE	− 13.18 (− 11.04^a^)	− 11.61	− 4.92	− 3.71
BTFE	− 12.70 (− 10.14^a^)	− 11.99	− 5.36	− 2.06
TTE	− 11.93 (− 9.56^a^)	− 12.05	− 6.29	− 2.38
B2E	− 7.87 (− 5.87^a^)	− 8.60	− 5.89	− 2.71
BPE	− 1.31 (0.14^a^)	− 2.85	− 1.22	− 2.00

^a^CCSD(T)/def2-TZVP (*ε* = 20.6) //M06-2X/def2-TZVP (*ε* = 20.6).

In addition, we investigated the fluorination effect of TMP solvent on the structural properties of electrolytes diluted with fluorinated-ether diluents. For this purpose, dimethyl(trifluoromethyl) phosphate (DTP) and tris(trifluoromethyl) phosphate (TTP) were considered as the partially and fully fluorinated TMP solvent, respectively (see Table 1). Similarly, the salt anions are mainly in aggregated states in diluted electrolytes based on DTP and TTP solvent (Fig. 1(d) and (g)), while the diluents are kept mainly in free state in DTP- and TTP-based electrolyte, respectively (Fig. 1(f) and (i)). However, we can observe clearly that the percentage of free state solvent increases as the degree of fluorination increases, while those of the CIP and aggregated states decrease (Fig. 1(b), (e) and (h)). QM calculations show that the interaction energy between DTP and Li^+^ is equal to − 18.57 kcal/mol, while that between TTP and Li^+^ is equal to − 15.98 kcal/mol (Supplementary Table S2). In fact, the interaction energy between Li^+^ and TMP, DTP or TTP solvent decreases as the degree of fluorination of TMP increases (Supplementary Table S2). Finally, it can be said that the concept of localized-concentrated electrolyte also stands in the diluted electrolytes based on partially as well as fully fluorinated TMP solvent. Such formation of localized aggregated salts is highly expected for the formation of a more compact and stable SEI layer brought about mainly from salt decomposition^9,10,12^.

**Figure 2:**
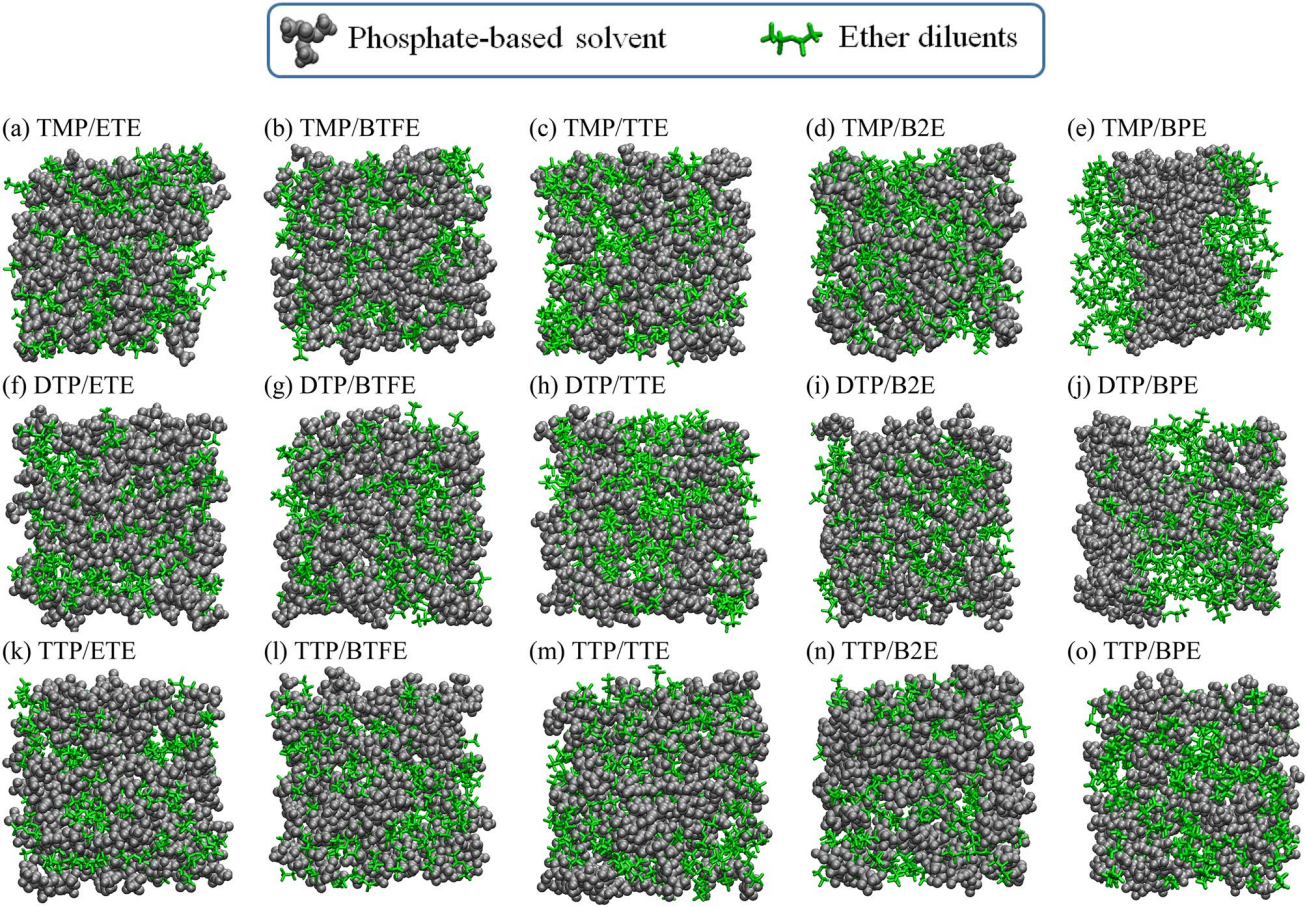
Snapshots of mixed solutions of (**a**) TMP/ETE, (**b**) TMP/BTFE, (**c**) TMP/TTE, (**d**) TMP/B2E, (**e**) TMP/BPE, (**f**) DTP/ETE, (**g**) DTP/BTFE, (**h**) DTP/TTE, (**i**) DTP/B2E, (**j**) DTP/BPE, (**k**) TTP/ETE, (**l**) TTP/BTFE, (**m**) TTP/TTE, (**n**) TTP/B2E and (**o**) TTP/BPE in equilibrium state. The considered molar ratio was 1solvent:1diluent.

Furthermore, we explored the miscibility of each diluent ether in pure TMP and LiFSA/TMP solutions. Based on the Flory–Huggins solution theory^18^, the Gibbs free energy (FE) change accompanying the mixing, Δ*G*_m_, was calculated as presented in Supplementary Table S3. For these calculations to evaluate the miscibility for each of 5 diluents, we prepared 5 systems of molecular ratio 1:1 of TMP/Diluent, and also each 5 systems of molecular ratio 1:1:1 and 1:1:2 of LiFSA/TMP/Diluent in LiFSA/TMP solution. In pure TMP solution, the hydrofluoroethers, viz ETE, BTFE, TTE and B2E were miscible as shown in Fig. 2a, 2b, 2c and 2d respectively. TTE showed higher miscibility with the largest Δ*G*_m_ equal to − 214.48 kcal/mol, while BTFE and B2E did comparatively good miscibility with the FEs of mixing equal to − 140.83 and − 119.17 kcal/mol, respectively. On the contrary, BPE showed an immiscible behavior in pure TMP solution (see Fig. 2e), with the positive Δ*G*_m_ equal to 53.09 kcal/mol. On the other hand, all ether diluents become miscible in LiFSA/TMP electrolyte solution as shown in Supplementary Fig. S1(a)–(e) with the highest and lowest miscibility for TTE and BPE, respectively. Moreover, Supplementary Table S3 showed that Δ*G*_m_ increases as the salt concentration increases, showing a higher miscible behavior of diluent ethers in higher salt concentration LiFSA/TMP/Diluent electrolyte solutions. Table 2 reported a set of interaction energies of ether diluents with electrolyte compounds as calculated at the QM level. We can know that the interaction energy of B2E with TMP solvent, − 5.89 kcal/mol, is larger than the mutual one with B2E themselves, − 2.00 kcal/mol. These results could explain the good miscibility behavior of B2E diluents on the TMP solvent (see Fig. 2(c)). Conversely, BPE showed a larger interaction between them, − 2.00 kcal/mol, than that with TMP solvent, − 1.22 kcal/mol, resulting in phase separation between TMP and BPE molecules as shown in Fig. 2(e). On the other hand, the interaction of diluent ethers with LiFSA was larger than that with TMP solvent or themselves leading to a higher miscible behavior of the diluent ethers by adding LiFSA salt in electrolyte solution. In addition, in Table 2, we can notice that the interaction of Li^+^ cation with diluents is weaker compared to that of TMP solvent. Accordingly, the present results were in very good agreement with the experimental data showing that the diluents were miscible with electrolyte solution but only weakly coordinated to Li^+^ compared to the solvent molecules^9,10,12,14,15^.

Furthermore, the fluorination effect of TMP solvent on the miscibility of the fluorinated-ethers diluents were investigated. Figure 2 (f), (g), (h) and (i) show that the hydrofluoroethers, viz ETE, BTFE, TTE and B2E were miscible in pure DTP solution, respectively, with larger Δ*G*_m_ in DTP than in TMP solution (Supplementary Tables S4 and S5). However, BPE shows a miscible behavior in DTP solution as illustrated in Fig. 2 (j), with a very small Δ*G*_m_ compared to those of the other hydrofluoroethers (Supplementary Tables S4). Also, in pure TTP solution, the tendency of miscibility is kept for 5 hydrofluoroethers as shown in Fig. 2 (k), (l), (m) and (n). The Δ*G*_m_ for each hydrofluoroether was the largest in pure TTP solution compared to the corresponding Δ*G*_m_ in DTP or TMP solution. It is similarly observed that BPE shows a miscible behavior in TTP solution as shown in Fig. 2 (o), with a larger Δ*G*_m_ in the same order to that of the other hydrofluoroethers (Supplementary Tables S4). In fact, the interaction energy between different phosphate-based solvents and fluorinated BPE increases as its degree of fluorination increases, which is regarded as a “fluorous effect”^22^. On the other hand, it is interesting to find that all the ether diluents become miscible by adding LiFSA in phosphate-based solvent solutions with considerable increase of Δ*G*_m_ (Supplementary Tables S4). The present results, therefore, clearly show that the miscibility of fluorinated ether diluent is significantly enhanced by increasing the degree of fluorination of TMP solvent, i.e., the “fluorous effect”^22^.

**Figure 3:**
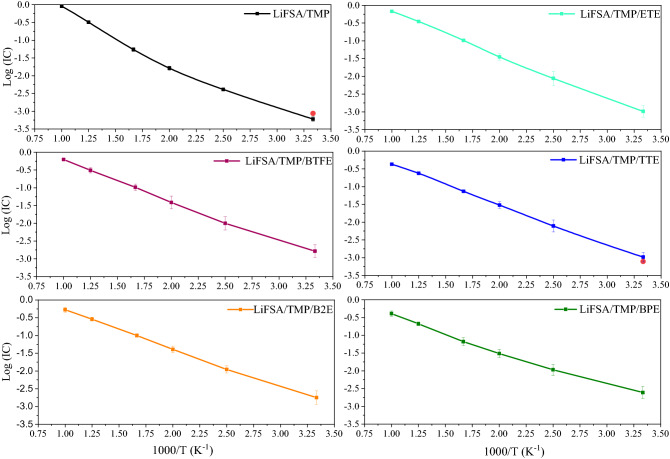
The calculated ionic conductivities as a function of temperatures for (**a**) HC and the diluted LiFSA/TMP based electrolyte by (**b**) ETE, (**c**) BTFE, (**d**) TTE, (**e**) B2E and (**f**) BPE, respectively. The red point presents the experimental value^10^.

**Figure 4:**
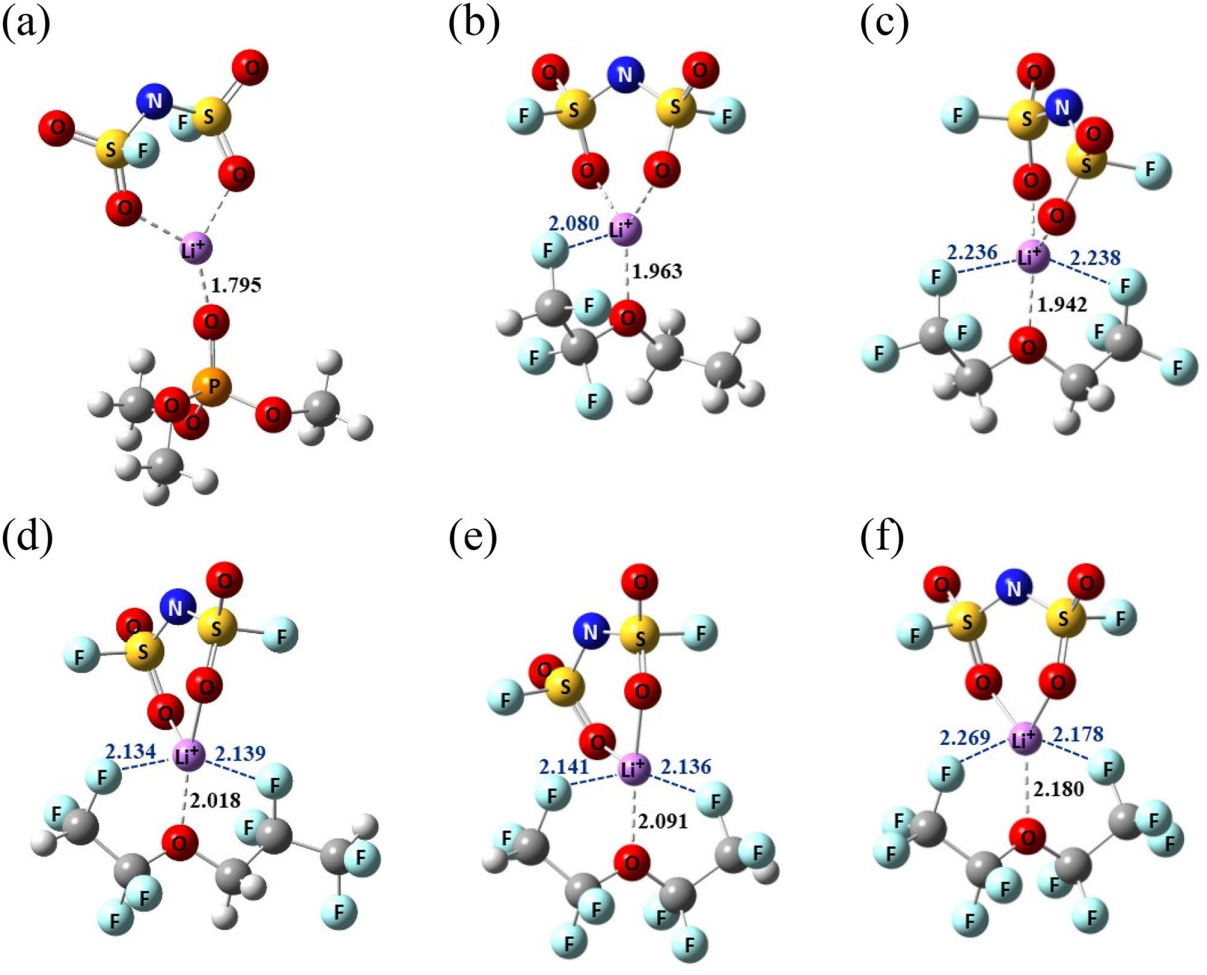
The optimized structure of (**a**) LiFSA-TMP, (**b**) LiFSA-ETE, (**c**) LiFSA-BTFE, (**d**) LiFSA-TTE, (**e**) LiFSA-B2E and (**f**) LiFSA-BPE obtained at M06-2X/def2-TZVP level in TMP (*ε* = 20.6) solvent. The selected distances are shown in Å.

Subsequently, the transport properties were evaluated using MD calculation over 10 samples for each system (see Computational details). Figure 3 showed the temperature-dependence of the ionic conductivity for HC electrolyte and different diluted electrolytes. We can notice an Arrhenius-like dependence of ionic conductivity in all systems with the activation energy about 2.71 kcal/mol for HC electrolyte and about 2.42, 2.22, 2.25, 2.13, and 1.89 kcal/mol for diluted electrolyte with ETE, BTFE, TTE, B2E, and BPE, respectively. Indeed, the activation energy was the lower in the diluted electrolyte with BPE and B2E among them. In Table 3, we reported the obtained values of the ionic conductivity of electrolyte as well as the self-diffusion constant of Li^+^ at 298 K. The ratio of self-diffusion constant of Li^+^ to that of FSA^-^ was around 1 (see Table 3), which confirms partially that FSA was mainly in aggregate state forming a localized-concentrated LiFSA/TMP based electrolyte^12^. Also, we can notice that the self-diffusion constant of Li^+^ and the ionic conductivity should increase with dilution by different ether molecules. Indeed, the averaged distance between aggregated LiFSA increased by adding diluent molecules (see Supplementary Fig. S2) forming the smaller clusters of aggregated LiFSA (see Supplementary Fig. S3), which should become easier to diffuse. The obtained ionic conductivity of HC electrolyte and the diluted electrolyte with TTE (Table 3), which are equal 0.604 ± 0.169 and 1.037 ± 0.184 mS cm^−1^ respectively, were found in good agreement with experimental values, 0.78 and 0.87 mS cm^−1^ in HC and diluted electrolyte by TTE, respectively^12^. The conductivity is relatively increased with the dilution by BTFE, while it is only slightly improved using ETE and TTE diluents. Remarkably, the transport properties should be significantly enhanced with the dilution by BPE and B2E showing the highest ionic conductivity and self-diffusion constant of Li^+^ in the former case. The obtained ionic conductivities of LiFSA/TMP electrolyte diluted by BPE and B2E are equal to 2.453 ± 0.299 and 1.794 ± 0.273 mS cm^−1^, respectively. It should be noteworthy that these values are comparable very much with the experimental ones of LiFSA/TMP diluted by TMP solvent, which were found about 2.77 mS cm^−1^ in 2.0 mol.L^−1^ LiFSA/TMP electrolyte^12^ and about 1.9 mS cm^−1^ in 3.0 mol.L^−1^ one^3,12^_._ To understand more precisely the microscopic effect of different diluents on the Li^+^ and FSA^-^ diffusion, we focused on the solvation structures between ether molecules and LiFSA. From Fig. 4, we can notice that the interatomic distance, between the Li^+^ and the oxygen atom(s) of ether molecules, is larger in B2E and BPE than in BTFE, ETE, and TTE cases. Such a larger distance should be the origin of the weaker interaction among them and LiFSA, leading to a higher diffusion of Li^+^ and FSA^-^ in the diluted electrolyte with BPE and B2E molecules.

**Table 3:** Transport properties of highly salt-concentrated electrolyte LiFSA/TMP system and 5 different diluted LiFSA/TMP based electrolyte systems at 298 K.

System	*D*_Li_ (× 10^–18^ m^2 ^s^−1^)	*D*_Li_/*D*_FSA_	*IC* (mS cm^−1^)
LiFSA/TMP	0.096 ± 0.015	0.954	0.604 ± 0.169 (0.78^*a*^)
LiFSA/TMP/ETE	1.075 ± 0.158	1.096	1.027 ± 0.196
LiFSA/TMP/BTFE	1.797 ± 0.177	1.023	1.646 ± 0.212
LiFSA/TMP/TTE	1.264 ± 0.135	0.929	1.037 ± 0.184 (0.87^a^)
LiFSA/TMP/B2E	2.035 ± 0.186	1.014	1.794 ± 0.273
LiFSA/TMP/BPE	3.022 ± 0.172	1.001	2.453 ± 0.299

^a^Ref.^12^.

## Conclusion

In conclusion, the present study has theoretically proposed a promising candidate of diluents with high degree of fluorination (Table 1) for nonflammable HC LiFSA/TMP electrolyte. The present diluents showed a miscibility behavior with a high ability to form a localized-concentrated electrolyte in HC LiFSA/TMP solution. The present study showed also that the miscibility behavior of each fluorinated ether diluent has been significantly enhanced as its degree of fluorination of TMP solvent increases, i.e., the “fluorous effect”. It is remarkable that the self-diffusion constant of Li^+^ and ionic conductivity should significantly be improved by dilution with bis(1,1,2,2-tetrafluoro ethyl) ether (B2E) and bis(pentafluoro ethyl) ether (BPE) due to their weaker interaction with LiFSA salt as well as TMP solvent, comparing to other considered HFEs. The ionic conductivity of LiFSA/TMP electrolyte diluted by BPE or B2E must be comparable with that by TMP solvent. Further experimental certification of this theoretical observation should be strongly encouraged to be done towards a realistic proposal with higher reliability. A collaborative work of theoretical and experimental studies must certainly reduce the cost and time of the experimentations and contribute in guiding the selection of the appropriate diluent co-solvent and the future practical application of such nonflammable electrolytes.

## Computational details

In this study, the generalized AMBER^23^ force field (GAFF)^24^ was used. The atomic charges were obtained with the Merz-Singh-Kollman (MSK)^25^ method by performing single-point QM calculation in gas phase for the optimized structure. In order to test the reliability of GAFF, the mass density of the pure diluent ethers was calculated by the NPT MD simulations at 1 atm and 298 K. The present system of HC LiFSA/TMP based electrolyte consists of 100 FSA^−^, 100 Li^+^ and 130 TMP molecules. To dilute this HC electrolyte, we added 200 molecules of diluent ethers.

To calculate the miscibility, we referred to the molar ratio of 1:1 and 1:1:2 for TMP:diluent and LiFSA:TMP:diluent systems. First, we mixed the systems at 1000 K during 1 ns, and relaxed them at 298 K during 10 ns. Then, we calculated the Gibbs free energy change^26^ that accompanies mixing:

1 $$\Delta G_{{\text{m}}} = KT\left[ {n_{{\text{s}}} {\text{ln}}\left( {\emptyset_{{\text{s}}} } \right) + n_{{\text{d}}} {\text{ln}}\left( {\emptyset_{{\text{d}}} } \right) + n_{{\text{s}}} \zeta_{{{\text{FH}}}} \emptyset_{{\text{d}}} } \right],$$

where, $n_{{\text{s}}}$ and $n_{{\text{d}}}$ are the number of molecules and $\emptyset_{{\text{s}}}$ and $\emptyset_{{\text{d}}}$ are the volume fractions of the solvent and diluents respectively. $\zeta_{{{\text{FH}}}}$ is the Flory–Huggins interaction parameter^18^, and is equal to:

1 $$\zeta_{{{\text{FH}}}} = \frac{{V_{{{\text{ref}}}} {\Delta }H_{{\text{m}}} 1}}{{KTV_{{\text{m}}} \emptyset_{{\text{s}}} \emptyset_{{\text{d}}} }},$$

where, $V_{{{\text{ref}}}}$ is the reference volume and equal to the volume of the smallest molecule. The change in enthalpy upon mixing per unit volume from the cohesive energy densities (*CED*)^27^ of pure solvent is expressed as follow:

3 $$\frac{{{\Delta }H_{{\text{m}}} }}{{V_{{\text{m}}} }} = CED_{{\text{m}}} - \left( {CED_{{\text{s}}} \emptyset_{{\text{s}}} + CED_{{\text{d}}} \emptyset_{{\text{d}}} } \right)$$

with

4 $$CED = \frac{\rho }{M}\left( {{\Delta }H_{{\text{v}}} - RT} \right).$$

The equations of enthalpy of vaporization and potential energy are expressed^28^ as follows:

5 $${\Delta }H_{{\text{v}}} = \left\langle {E_{{{\text{tot}},{\text{bulk}}}} - E_{{{\text{tot}},{\text{molecules}}}} } \right\rangle + RT$$

and

6 $$E_{{{\text{tot}}}} = E_{{{\text{bonded}}}} + E_{{{\text{nonbonded}}}} .$$

For the calculation of the self-diffusion constant and the ionic conductivity, we performed NVT MD simulations at 900, 800, 600, 500, 450, and 300 K during 50 ns. We first calculated the self-diffusion constant using the Einstein equation^29^:

7 $$6Dt = r^{2} \left( t \right).$$

Then, the ionic conductivity was calculated using Nernst–Einstein equation^30^:

8 $$\sigma = \frac{{N_{{\text{A}}} e^{2} z^{2} \left( {\left[ {C_{{{\text{Li}}^{ + } }} D_{{{\text{Li}}^{ + } }} } \right] + \left[ {C_{{{\text{FSA}}^{ - } }} D_{{{\text{FSA}}^{ - } }} } \right]} \right)}}{{k_{{\text{B}}} T}},$$

where *z* is the charge, *C* and *D* are the concentration and the self-diffusion constant of anions. *k*_B_ is the Boltzmann constant and *T* is the absolute temperature.

In order to fit the ionic conductivity as a function as the inverse of the temperature according to the Arrhenius behavior, we use the following equation^31^:

9 $$IC = IC_{0} e^{{ - \frac{{E_{{\text{a}}} }}{{k_{{\text{B}}} T}}}} ,$$

where the activation energy for the ionic conductivity (*E*_a_) and the exponential factor (*IC*_0_) are both temperature independent variables.

All the QM calculations were calculated with Gaussian09 program^32^. The geometry optimizations and frequency calculations have been done at M06-2X/def2-TZVP level of theory using the solvation model based on density (SMD) with the dielectric constant of 20.6, which corresponds to that of the TMP solvent. We mentioned this methodology as the QM level. The several different configurations were considered and selected the energetically most preferred one (see Supplementary Figs. S4–S9). The interaction energy for the system, AB ($E_{{{\text{AB}}}}^{{{\text{Int}}}}$), were calculated as:

10 $$E_{{{\text{AB}}}}^{{{\text{Int}}}} = E_{{{\text{AB}}}}^{{{\text{Opt}}}} - E_{{\text{A}}}^{{{\text{Opt}}}} - E_{{\text{B}}}^{{{\text{Opt}}}}$$

where $E_{{{\text{AB}}}}^{{{\text{Opt}}}}$, $E_{{\text{A}}}^{{{\text{Opt}}}}$ and $E_{{\text{B}}}^{{{\text{Opt}}}}$ are the optimized energy values for AB, A, and B systems, respectively. The interaction energy between Li^+^ and the TMP solvent (or ether diluents) was (were) also calculated by performing single point calculations at CCSD(T)/def2-TZVP level of theory with dielectric constant of 20.6 after taking the optimized geometry from the M06-2X/def2-TZVP level with dielectric constant of 20.6. We have noticed that M06-2X method can produce a similar interaction energy trend as CCSD(T) with a decent computational cost (see Table 2). The energy value for Li^+^ in TMP solvent is − 7.421205 a.u. at M06-2X/def2-TZVP whereas that of at CCSD(T)/def2-TZVP is − 7.374694 a.u.

## Supplementary information

Supplementary Information.
