# Supplementary material for: Theoretically predicting the feasibility of highly-fluorinated ethers as promising diluents for non-flammable concentrated electrolytes
Source: Sci Rep. 2020 Dec 15;10:21966. doi: 10.1038/s41598-020-79038-y (PMC7738504; doi:10.1038/s41598-020-79038-y)
Supplement: Supplementary file 1 — Supplementary Information. [file 41598_2020_79038_MOESM1_ESM.pdf]

Supplementary Material

Theoretically Predicting the Feasibility of Highly-Fluorinated Ethers as Promising Diluents for Non-flammable Concentrated Electrolytes

Amine Bouibes[1], Soumen Saha[1, 2] & Masataka Nagaoka[1, 2 *]

[1]Graduate School of Informatics, Nagoya University, Furo-cho, Chikusa-ku, Nagoya 464-8601, Japan

[2]Elements Strategy Initiative for Catalysts and Batteries (ESICB), Kyoto University, Kyodai Katsura, Nishikyo-ku, Kyoto 615-8520, Japan;

***Correspondence to:** mnagaoka@i.nagoya-u.ac.jp

Table S1. Molar ratios and salt concentrations of highly salt-concentrated electrolyte LiFSA/TMP system and 5 different diluted LiFSA/TMP based electrolytes.

System	Molar ratio	Salt concentration ($mol.L^{-1}$)
LiFSA/TMP	1:1.3 (HC)	4.33 (4.10[a])
LiFSA/TMP/ETE	1:1.3:2 (diluted)	2.18
LiFSA/TMP/BTFE	1:1.3:2 (diluted)	2.07
LiFSA/TMP/TTE	1:1.3:2 (diluted)	1.88 (1.80[a])
LiFSA/TMP/B2E	1:1.3:2 (diluted)	1.98
LiFSA/TMP/BPE	1:1.3:2 (diluted)	1.86

[a]Takada, K.; Yamada, Y.; Yamada, A. *ACS Appl. Mater. Interfaces* **2019**, *11*, 35770−35776

Table S2: The interaction energy (kcal/mol) between Li^+, LiFSA and TMP with DTP and TTP systems as well as the interaction energy (kcal/mol) between the diluent themselves (dimer) as obtained at M06-2X/def2-TZVP level in TMP (ε = 20.6) solvent.

System	Li^+-System	LiFSA- System	TMP- System	Dimer
**DTP**	-18.57	-15.74	-4.82	-5.30
**TTP**	-15.98	-13.89	-6.19	-5.43

Table S3. The calculated Gibbs free energy changes accompanying the mixing ΔG_m in the TMP/Diluent solution and the LiFSA/TMP/Diluent electrolyte solutions diluted with 5 different ether molecules (Diluents) at 2 different molar ratios of LiFSA/TMP/Diluent.

	ΔG_m (kcal/mol)		
**Molar ratio**	**TMP/Diluent** 1:1	**LiFSA/TMP/Diluent** 1:1:1	**LiFSA/TMP/Diluent** 2:1:1
**ETE**	-86.57	-871.70	-12383.39
**BTFE**	-140.83	-1181.66	-13306.39
**TTE**	-214.48	-1213.15	-14351.81
**B2E**	-119.17	-723.78	-12442.90
**BPE**	+53.09	-531.15	-11215.57

Table S4. The calculated Gibbs free energy changes accompanying the mixing ΔG_m in the DTP/Diluent and TTP/Diluent solutions and the LiFSA/DTP/Diluent and LiFSA/TTP/Diluent electrolyte solutions diluted with 5 different ethers molecules (Diluents).

	**ΔG_m (kcal/mol)**			
	**DTP/Diluent 1:1**	**LiFSA/DTP/Diluent 1:1:1**	**TTP/Diluent 1:1**	**LiFSA/TTP/Diluent 1:1:1**
**ETE**	-4366.66	-11564.79	-15783.05	-24922.01
**BTFE**	-4468.1	-13481.61	-16005.46	-28431.54
**TTE**	-4719.68	-17401.44	-16201.01	-32680.73
**B2E**	-4432.66	-13217.28	-15883.31	-26095.28
**BPE**	-1233.11	-10071.34	-15985.24	-28153.98

Table S5. The calculated interaction energies (E_{inter}) using MD calculations for 3 molecular systems of TMP/BPE, DTP/BPE and TTP/BPE solutions, each of which consists of both components of 200 molecules.

**System**	**Molar ratio**	**E_{inter} (kcal/mol)**
TMP/BPE	1:1	-342.77
DTP/BPE	1:1	-812.62
TTP/BPE	1:1	-947.17

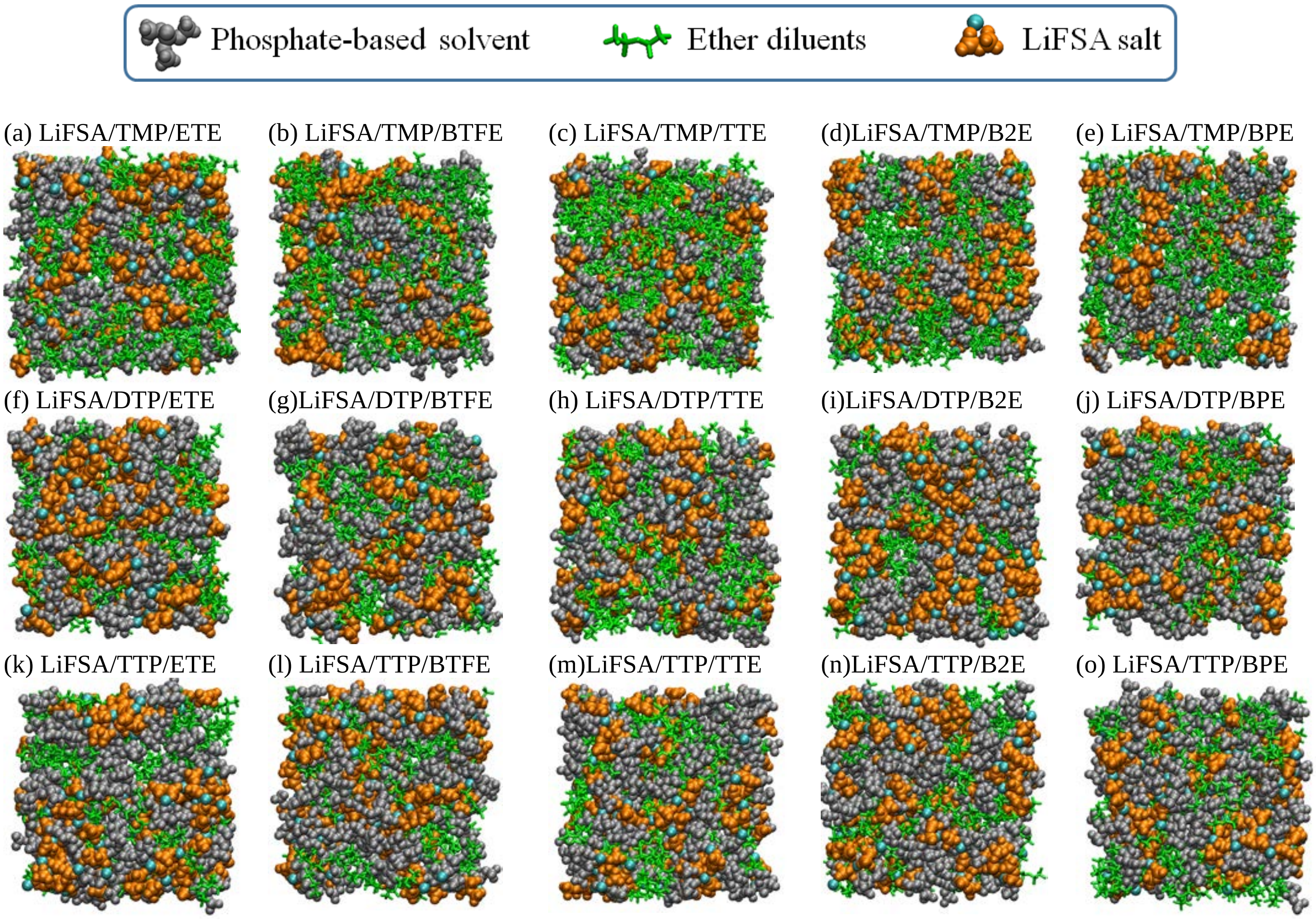

Figure S1. Snapshots of mixed solutions of (a) LiFSA/TMP/ETE, (b) LiFSA/TMP/BTFE, (c) LiFSA/TMP/TTE, (d) LiFSA/TMP/B2E, (e) LiFSA/TMP/BPE, (f) LiFSA/DTP/ETE, (g) LiFSA/DTP/BTFE, (h) LiFSA/DTP/TTE, (i) LiFSA/DTP/B2E, (j) LiFSA/DTP/BPE, (k) LiFSA/TTP/ETE, (l) LiFSA/TTP/BTFE, (m) LiFSA/TTP/TTE, (n) LiFSA/TTP/B2E and (o) LiFSA/TTP/BPE in equilibrium state.

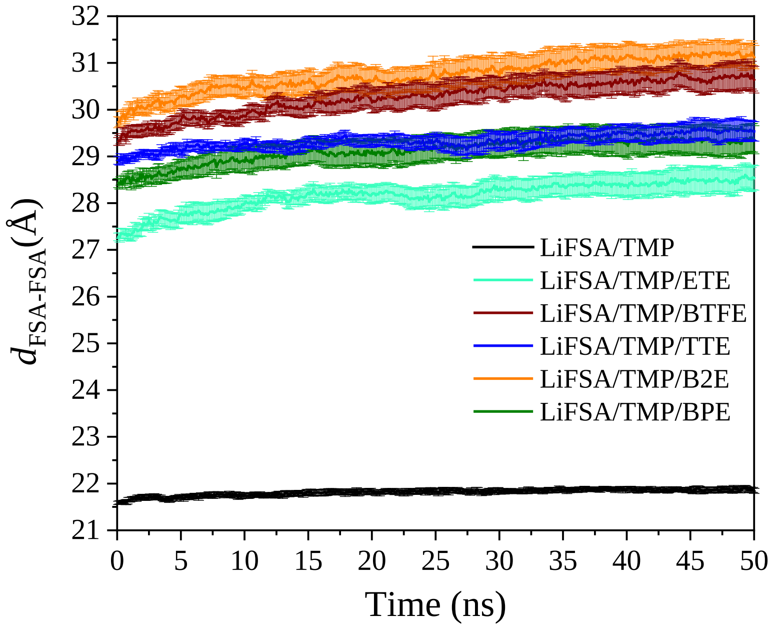

Figure S2. Averaged distance between FSA- anions in HC and different diluted LiFSA/TMP based electrolytes.

a) b)

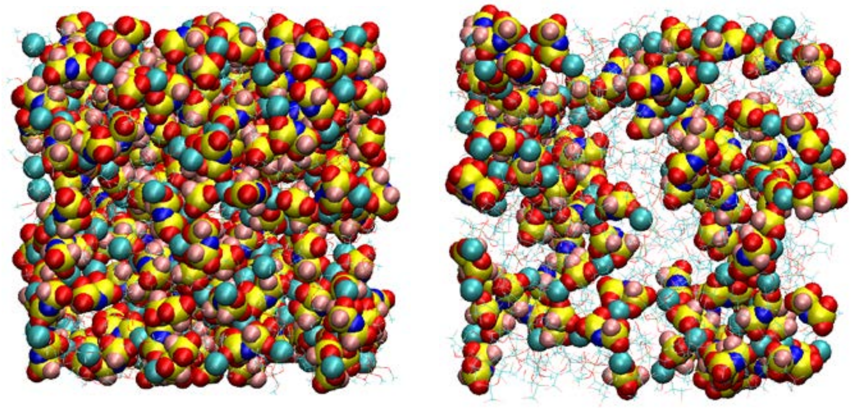

Figure S3. Typical snapshot of aggregated salt in HC and diluted LiFSA/TMP based electrolyte.

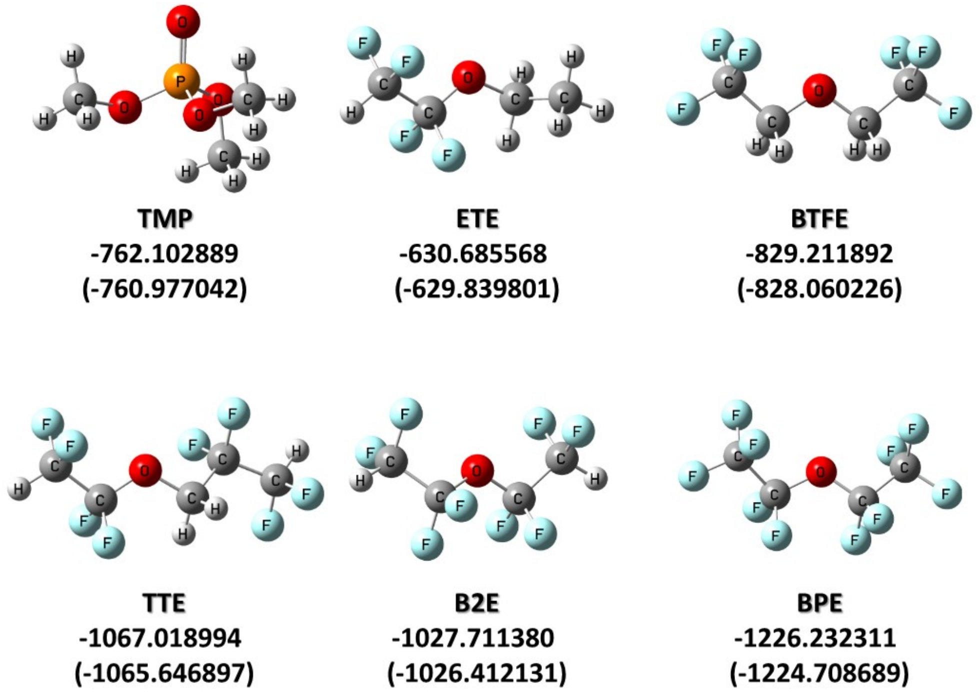

Figure S4. Optimized geometries of the considered solvent and diluents as obtained at M06-2X/def2-TZVP level of theory with dielectric constant of 20.6. The obtained electronic energy values (a.u.) at M06-2X/def2-TZVP are mentioned. In parenthesis, CCSD(T)/def2-TZVP energy values (a.u.) are also mentioned.

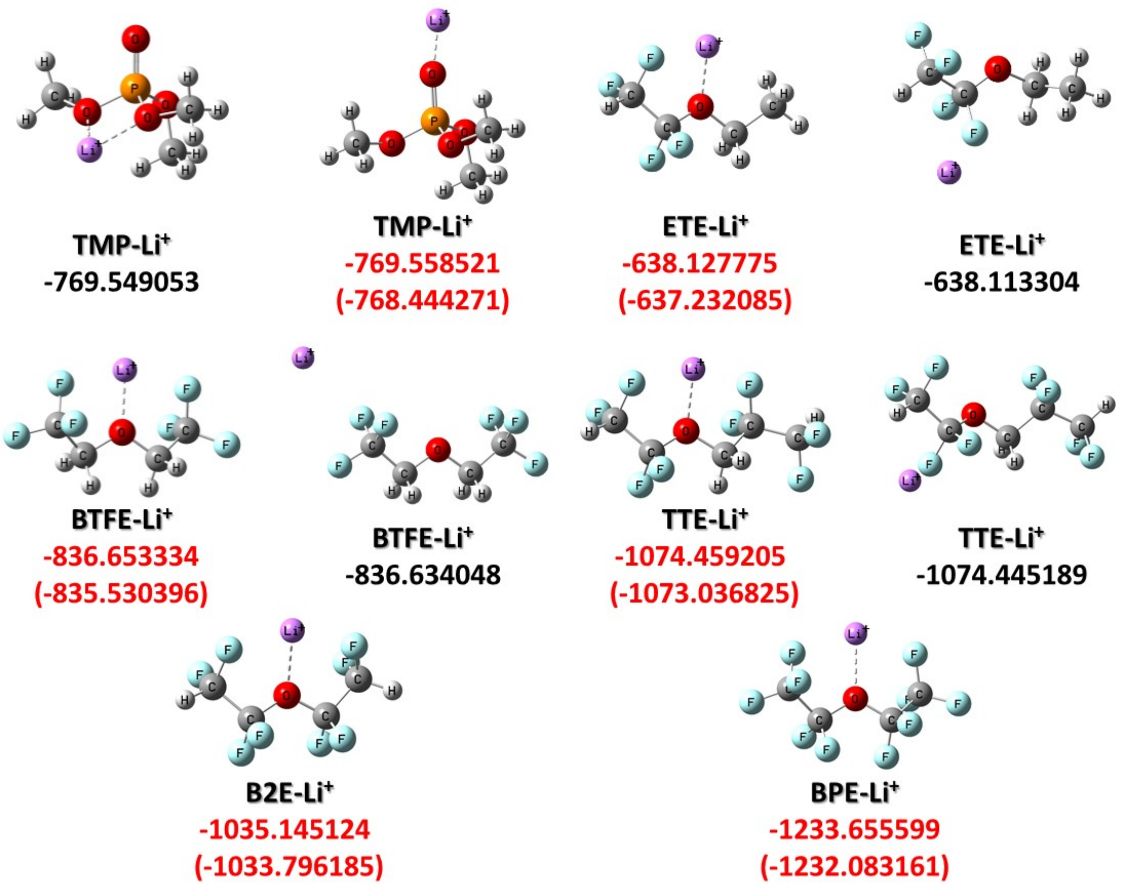

Figure S5. Optimized geometries of various Li^+-complexes as obtained at M06-2X/def2-TZVP level of theory with dielectric constant of 20.6. The obtained electronic energy values (a.u.) at M06-2X/def2-TZVP are mentioned. The most preferable energy values are shown in red color for each complex. In parenthesis, CCSD(T)/def2-TZVP energy values (a.u.) for most preferable Li^+-complexes are also mentioned.

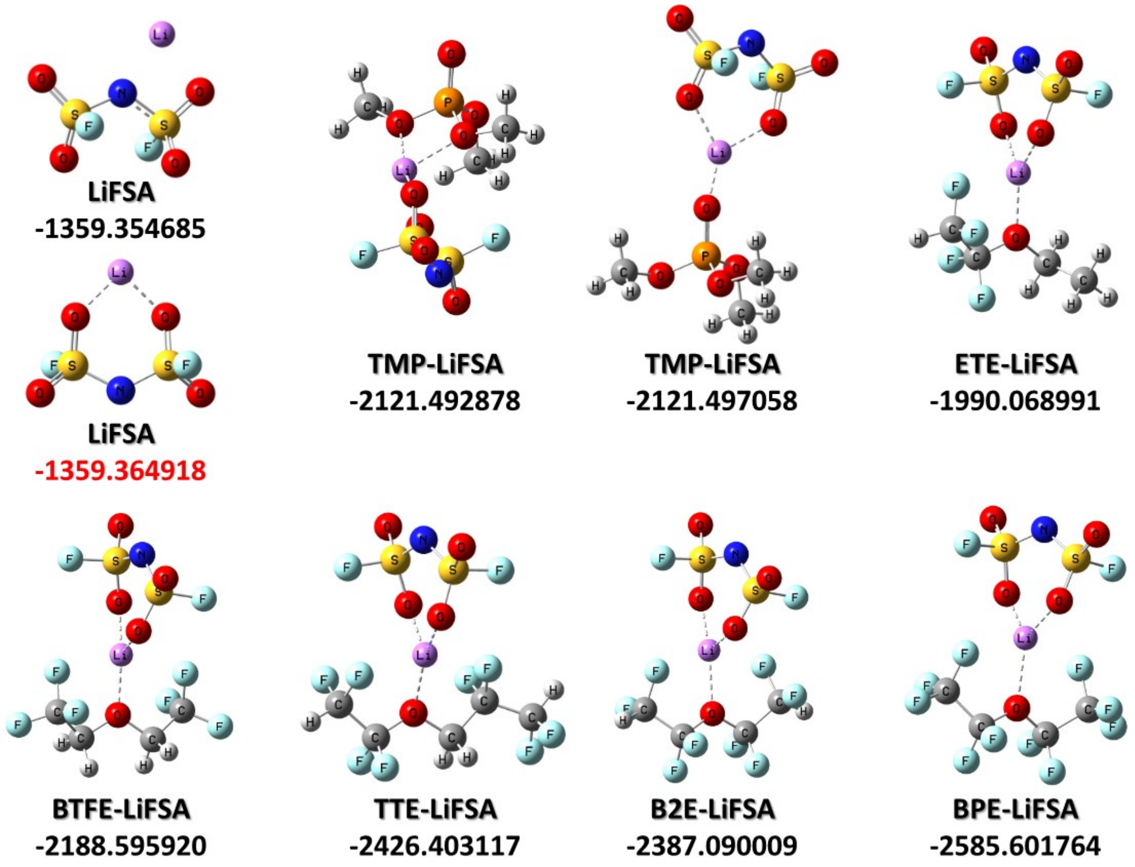

Figure S6. Optimized geometries of various LiFSA and LiFSA-complexes as obtained at M06-2X/def2-TZVP level of theory with dielectric constant of 20.6. The obtained electronic energy values (a.u.) at M06-2X/def2-TZVP are mentioned. The most preferable energy value for LiFSA is shown in red color.

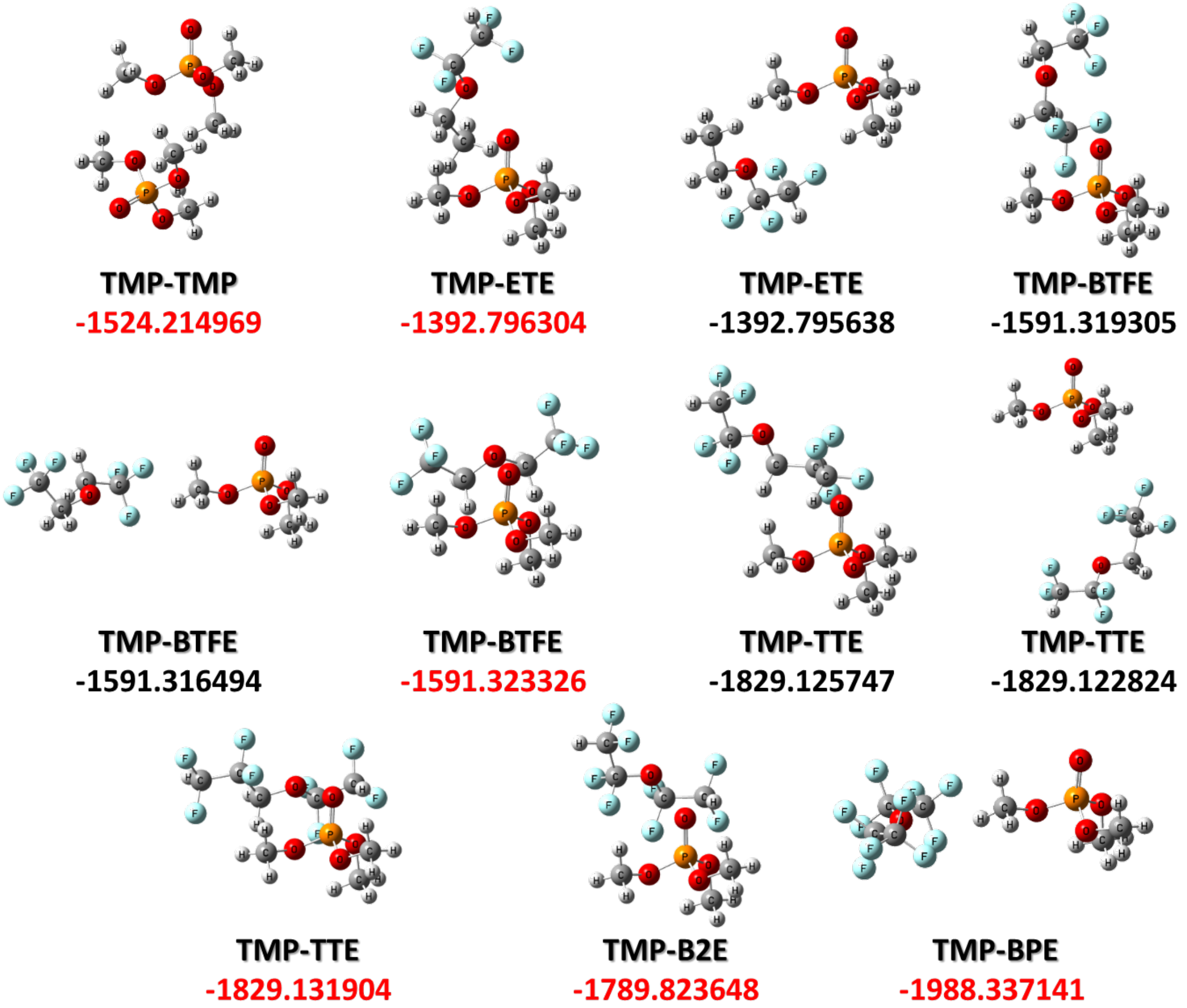

Figure S7. Optimized geometries of various TMP-complexes as obtained at M06-2X/def2-TZVP level of theory with dielectric constant of 20.6. The obtained electronic energy values (a.u.) at M06-2X/def2-TZVP are mentioned. The most preferable energy values are shown in red color for each complex.

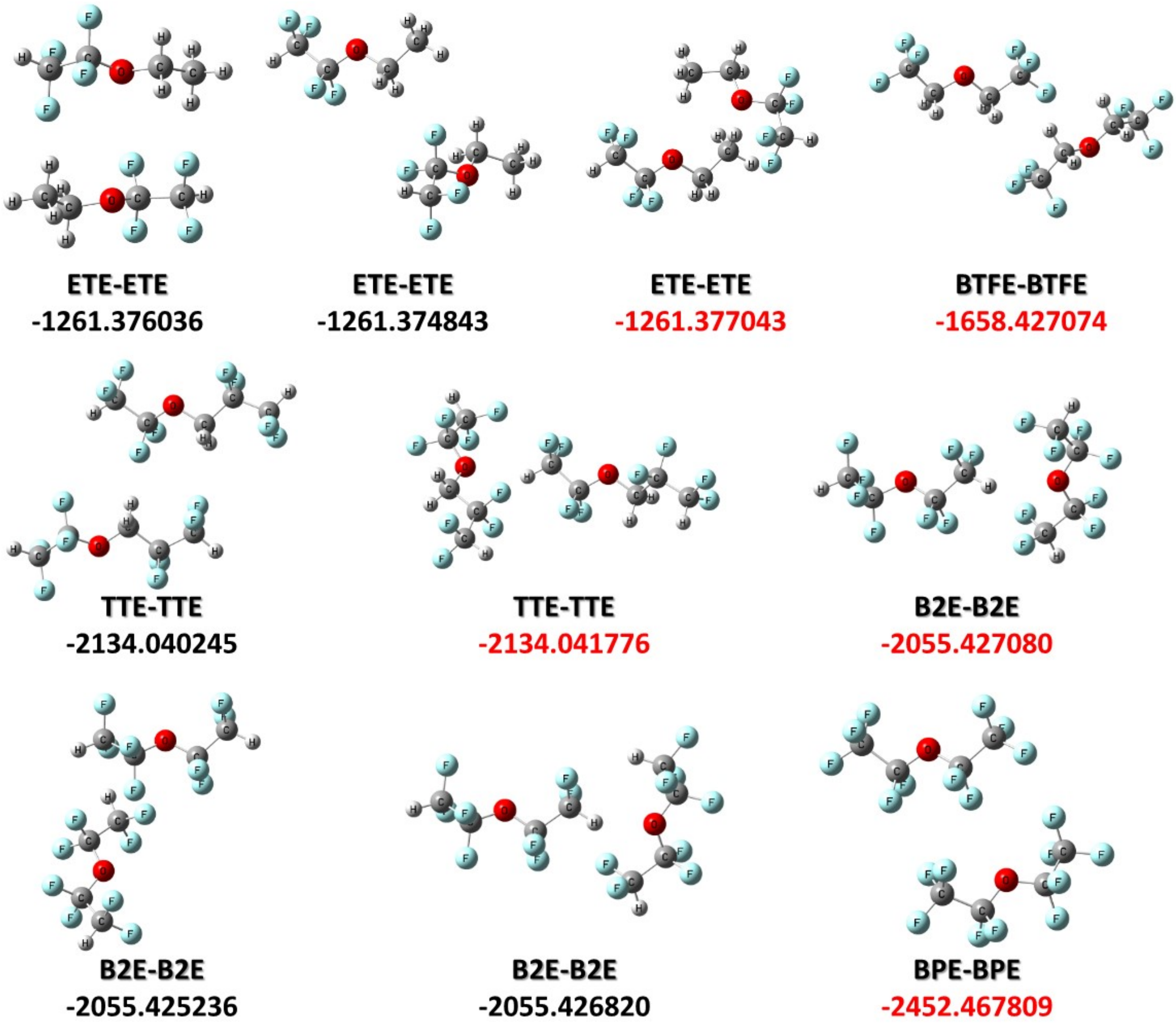

Figure S8. Optimized geometries of various diluent-diluent dimer complexes as obtained at M06-2X/def2-TZVP level of theory with dielectric constant of 20.6. The obtained electronic energy values (a.u.) at M06-2X/def2-TZVP are mentioned. The most preferable energy values are shown in red color for each complex.

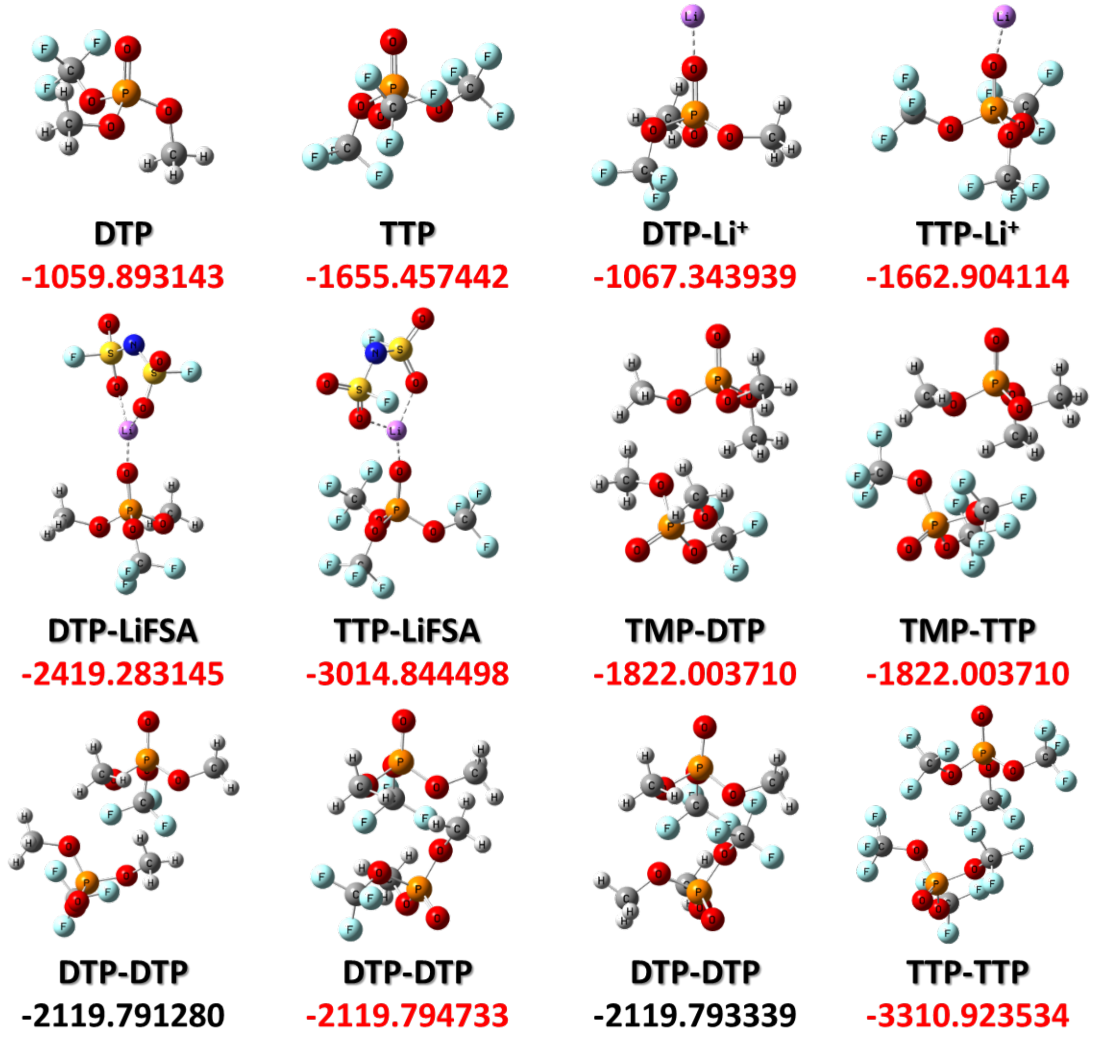

Figure S9. Optimized geometries of various structures for DTP and TTP as obtained at M06-2X/def2-TZVP level of theory with dielectric constant of 20.6. The obtained electronic energy values (a.u.) at M06-2X/def2-TZVP are mentioned. The most preferable energy values are shown in red color.
